# Probing the role of PPARγ in the regulation of late-onset Alzheimer’s disease-associated genes

**DOI:** 10.1371/journal.pone.0196943

**Published:** 2018-05-03

**Authors:** Julio Barrera, Shobana Subramanian, Ornit Chiba-Falek

**Affiliations:** 1 Department of Neurology, Duke University Medical Center, Durham, North Carolina, United States of America; 2 Center for Genomic and Computational Biology, Duke University Medical Center, Durham, North Carolina, United States of America; Taipei Veterans General Hospital, TAIWAN

## Abstract

Peroxisome proliferator-activated receptor-γ (PPARγ), is a transcription factor that governs pathways, such as lipid metabolism and immune response, that have been implicated in the etiology of LOAD. Previously, we established HepG2-derived cell-lines with stable knockdown of PPARγ gene, and showed an increase in mRNA levels of genes mapped in the *APOE* linkage disequilibrium (LD) region on chromosome 19q13.32, with the greatest effect observed for *APOE*-mRNA. Here, we extended the analysis using our *PPARγ* knockdown model system and investigated the broader effect on expression changes of genes implicated in LOAD via genome wide association studies (GWAS). We applied the nCounter gene expression assay (NanoString) using a panel of twenty-four LOAD-associated genes inferred by proximity to the top significantly associated SNPs. Two independent *PPARγ* knockdown cell-lines showed changes in mRNA levels of a total of seven genes compared to a control HepG2 cell-line; six of which, *ABCA7*, *APOE*, *CASS4*, *CELF1*, *PTK2B*, and *ZCWPW1*, were upregulated and one, *DSG2*, was downregulated upon *PPARγ* knockdown. Our results propose that *PPARγ* may act as a master regulator of the transcription of several genes involved in LOAD pathogenesis. Our study provided the premise for further analyses including a larger set of genes positioned within a wider range of linkage disequilibrium (LD) regions tagged by all LOAD significantly associated SNPs.

## Introduction

Large multi-center genome-wide association studies (GWAS) found associations between late-onset Alzheimer’s disease (LOAD) and over twenty genomic loci [1–6]. Subsequent studies have mapped pathways on which the genes within LOAD-associated regions participate, and identified diverse biological pathways, including lipid metabolism, immune and inflammatory response, and endocytosis [7–9]. The involvement of various pathways supports the concept of LOAD as a system-wide disorder. However, the molecular mechanisms through which the LOAD-associated loci exert their pathogenic effects remain to be fully elucidated.

It has been suggested that alteration in the levels of normal (wild-type) genes that are important in maintaining normal brain function can lead to neurodegenerative diseases, including LOAD [10–13]. Furthermore, expression quantitative trait loci (eQTLs) within LOAD-associated regions were described in brain regions vulnerable to LOAD [14, 15]. These studies strengthened the important role of the regulation of gene expression in LOAD etiology. Thus, it is imperative to better understand the mechanisms such as transcription regulation, that mediate the expression levels of the LOAD-associated genes.

The ligand-activated nuclear transcription factor, peroxisome proliferator-activated receptor-γ (PPARγ), has been shown to regulate the transcription of numerous genes playing key roles in adipocyte differentiation, inflammation and immune response, insulin sensitivity, and lipid and glucose metabolism [16–18]. Intriguingly, PPARγ governed pathways overlap, to some extent, with the biological pathways implicated in LOAD pathogenesis via GWAS, epidemiological studies, and other evidence [7–9, 19].

The *APOE* linkage disequilibrium (LD) region on 19q13.32 is the strongest genetic risk factor for LOAD [20–31]. Recently, using the short hairpin RNA (shRNA) method in HepG2 cells, we measured the effects of *PPAR*γ knockdown on mRNA expression of genes within the chr19q13.32 region, and demonstrated increases in the levels of *TOMM40-*, *APOE-*, and *APOC1*-mRNAs; *APOE*-mRNA was the most responsive, showing a 50% increase in expression relative to control [32]. As a complementary approach, we applied PPARγ agonists and demonstrated that PPARγ activation decreased the levels of all three transcripts, with the strongest effect on *APOE*-mRNA as well [32]. These observations further established a role for PPARγ in the transcriptional regulation of the most significant LOAD genetic risk factor. The intersection between biological processes regulated by PPARγ and those involved in LOAD pathogenesis may be mediated through a master role of PPARγ in the transcriptional modulation of additional LOAD-associated genes. Here, we extended our previous analysis to other genes implicated in LOAD, and focused on the genes inferred by proximity to the LOAD-associated SNPs (Table 1). We applied NanoString technology to characterize the effect of PPARγ knockdown on the expression of twenty-four LOAD-GWAS genes involved in various physiological pathways.

**Table 1:** LOAD-risk genes located ± 100 kb of the top SNP.

Closest gene	Top SNP*	Chr.	Position**	OR (95% CI)	*P* value	mRNA expression HepG2
CR1	rs6656401	1	207,692,049	1.18 (1.14–1.22)	5.7×10^−24^	n.a.
BIN1	rs6733839	2	127,892,810	1.22 (1.18–1.25)	6.9×10^−44^	+
INPP5D	rs35349669	2	234,068,476	1.08 (1.05–1.11)	3.2×10^−8^	++
MEF2C	rs190982	5	88,223,420	0.93 (0.90–0.95)	3.2×10^−8^	n.a.
CD2AP	rs10948363	6	47,487,762	1.10 (1.07–1.13)	5.2×10^−11^	+++
TREM2/TREML2	rs9381040	6	41,154,650	0.93 (0.91–0.96)	6.3×10^−7^	n.a.
HLA-DRB5–HLA-DRB1	rs9271192	6	32,578,530	1.11 (1.08–1.15)	2.9×10^−12^	n.a.
NME8	rs2718058	7	37,841,534	0.93 (0.90–0.95)	4.8×10^−9^	n.a.
ZCWPW1	rs1476679	7	100,004,446	0.91 (0.89–0.94)	5.6×10^−10^	+
EPHA1	rs11771145	7	143,110,762	0.90 (0.88–0.93)	1.1×10^−13^	+
PTK2B	rs28834970	8	27,195,121	1.10 (1.08–1.13)	7.4×10^−14^	+
CLU	rs9331896	8	27,467,686	0.86 (0.84–0.89)	2.8×10^−25^	n.a.
CELF1	rs10838725	11	47,557,871	1.08 (1.05–1.11)	1.1×10^−8^	++
MS4A6A-MS4A1	rs983392	11	59,923,508	0.90 (0.87–0.92)	6.1×10^−16^	n.a.
PICALM	rs10792832	11	85,867,875	0.87 (0.85–0.89)	9.3×10^−26^	++
SORL1	rs11218343	11	121,435,587	0.77 (0.72–0.82)	9.7×10^−15^	++
FERMT2	rs17125944	14	53,400,629	1.14 (1.09–1.19)	7.9×10^−9^	++
SLC24A4-RIN3	rs10498633	14	92,926,952	0.91 (0.88–0.94)	5.5×10^−9^	n.a.
DSG2	rs8093731	18	29,088,958	0.73 (0.62–0.86)	1.0×10^−4^	++
ABCA7	rs4147929	19	1,063,443	1.15 (1.11–1.19)	1.1×10^−15^	+
APOE^#^	rs4420638	19	45,422,945	3.58 (3.37–3.80)	1.1×10^−300^	+++
CD33	rs3865444	19	51,727,962	0.94 (0.91–0.96)	3.0×10^−6^	n.a.
CASS4	rs7274581	20	55,018,260	0.88 (0.84–0.92)	2.5×10^−8^	+

Adapted from the largest meta analysis (Lambert et al, 2013).

*SNPs showing the best level of association after meta-analysis of stages 1 and 2;

**Build 37/ hg19;

^#^Naj et al (2011); relative mRNA expression levels in HepG2 derived cell-lines: n.a.’ not detected; +, low; ++, intermediate; +++, high

## Materials and methods

### Cell lines

HepG2 derived cell-lines with stable PPARγ knockdown were generated by lentiviral shRNA transduction as previously described [32]. Two clones ID TRCN0000001673 (PPARγ 1673) [32] and ID TRCN0000001674 (PPARγ 1674), were selected for further analysis. The PPARγ knockdown cell-lines are referred as ‘PPARγ-KD1’ and ‘PPARγ-KD2’, respectively. To control for the effect of viral transduction and general shRNA expression, we used a control HepG2 derived cell line expressing shRNA targeting green fluorescent protein (GFP) as previously described [32], hereafter referred as ‘GFP’. In addition, we used the control un-transduced HepG2 cell-line, hereafter referred as ‘U’. The efficiency of PPARγ knockdown at the RNA level was previously validated by quantitative reverse transcription PCR (qRT-PCR) using the TaqMan system [32].

### Cell culture

The transduced HepG2 cell-lines were cultured in Eagle's minimum essential medium (MEM), supplemented with 10% fetal bovine serum, 2 mM GlutaMAX, 1 mM sodium pyruvate, 0.1 mM non-essential amino acids, penicillin streptomycin (10,000 units/mL penicillin and 10,000 μg/mL streptomycin), and puromycin dihydrochloride (2 μg/mL). Cells were maintained in a humidified incubator at 37 °C and 5% CO2.

Four HepG2 derived cell-lines were studied: PPARγ-KD1, PPARγ-KD2, GFP, and un-transduced. For each HepG2 derived cell-line 1.5 × 105 cells were plated onto each well of a 6-well plate and were cultured for thirty-six hours in puromycin-free media prior to harvesting for molecular evaluations. We repeated this experiment four times for each HepG2 derived cell-line, *i*.*e*., cells were cultured for thirty-six hours and harvested in four independent experiments.

### RNA extraction and sample preparation

Total RNA was extracted from cells using TRIzol reagent (Invitrogen, Carlsbad, CA), and purified using RNeasy Mini Kits (QIAGEN, Valencia, CA) according to the manufacturer's protocol. The concentration of RNA samples was determined spectrophotometrically by NanoDrop, and the quality of the RNA and lack of significant degradation was confirmed utilizing an Agilent Bioanalyzer. For all samples used, the RNA Integrity Number (RIN) was greater than eight, considered high quality RNA.

For each HepG2 derived cell-line, RNA was extracted from four independent experiments, and then was pooled such that each RNA sample represents four independent experiments (*i*.*e*., four biological replicates) for a particular HepG2 derived cell-line.

### NanoString nCounter gene expression analysis

Gene expression was quantified digitally using the nCounter Gene Expression Assay (NanoString Technologies, Seattle, WA). We developed a custom probe set termed CodeSet containing reporter and capture probes for twenty-five target genes and three housekeeping genes (*GAPDH*, *B2M*, and *LDHA*) (S1 Table). The CodeSet was designed and validated by NanoString such that each target-specific probe would cover all known transcript isoforms of a particular gene and (each probe sequence provided in S1 Table). Assay was performed using the NanoString protocols according to the manufacturer’s instructions. Briefly, for each pooled RNA sample 100ng was hybridized to the CodeSet overnight at 65°C. The hybridized samples underwent automated processing on the nCounter Prep Station, nCounter Master Kit reagents were added to remove the excess probes, and the purified target/probe complexes were immobilized in the nCounter cartridge for data collection. Data collection for digital quantification was carried out in the nCounter Digital Analyzer by processing the digital images of the color-coded barcodes on the surface of the cartridge, and tabulating the barcode counts for each target mRNA in each sample. The raw counts expression data was analyzed using nSolver Analysis Software (NanoString). Briefly, we normalized samples according to six positive and eight negative control probes and the geometric mean of the three housekeeping genes. For each sample, the background threshold was set using the geometric mean of the eight negative control probes plus two standard deviations, and the background subtraction was performed. Next, data was normalized using the geometric mean of the six positive control probes and three housekeeping genes as follows: The normalization factor for each sample was calculated using the geometric mean of the six positive control probes, specifically by dividing the arithmetic mean of the geometric mean by the geometric mean value. This was followed by a technical normalization using the geometric mean of the three housekeeping genes included in each run (*GAPDH*, *B2M*, and *LDHA*). The normalized data was log2 transformed and fold expression changes were calculated.

## Results

**Fig 1:**
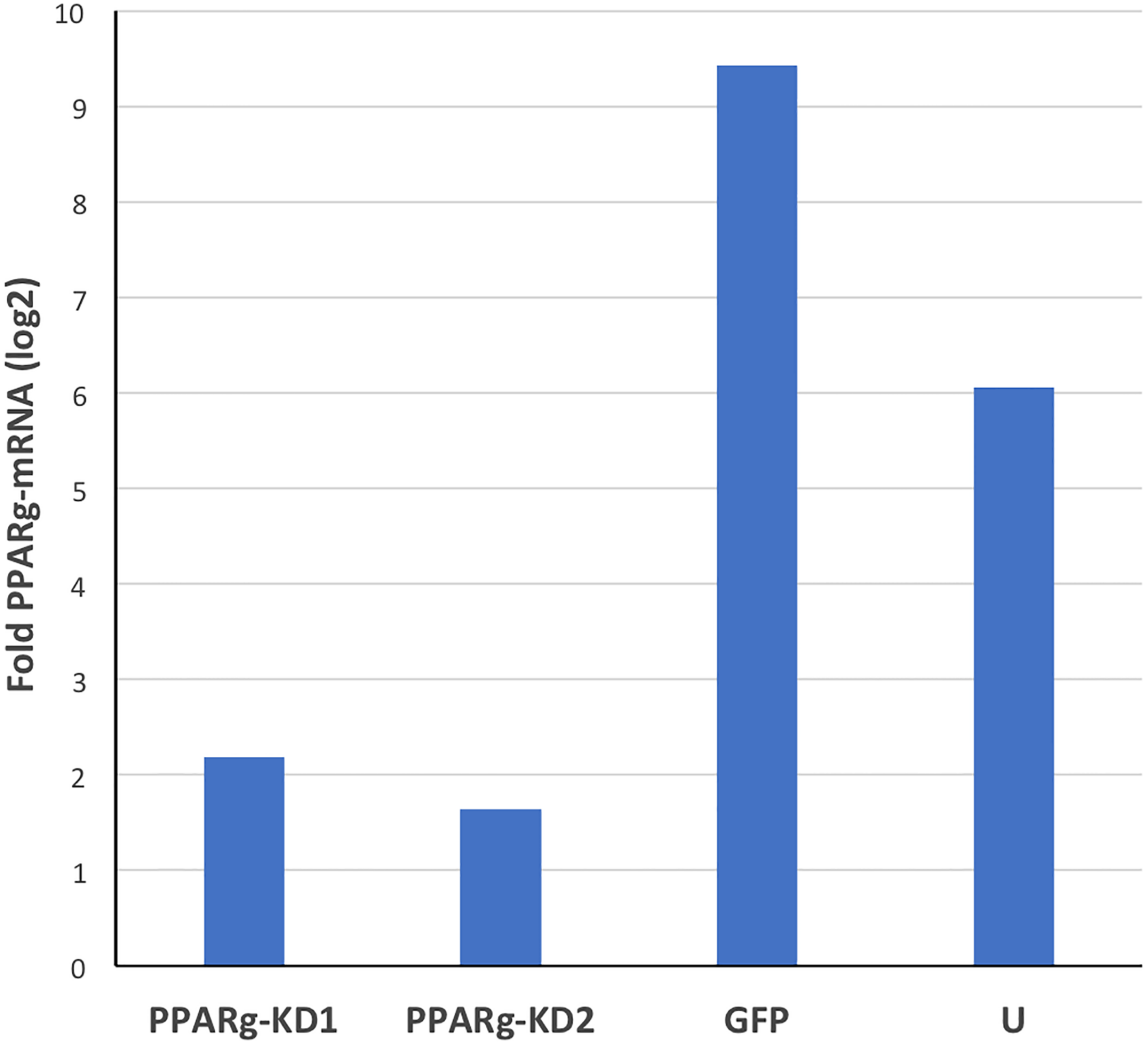
Validation of the reduction in *PPARγ*-mRNA expression in HepG2-derived *PPARγ*-KD1 and -KD2 cells. RNA was extracted from four HepG2 derived cell-lines: PPARγ-KD1, PPARγ-KD2, GFP, and untransduced (U). For each cell-line, RNA samples from four biological replicates were pooled. The levels of *PPARγ*-mRNA relative to the geometric mean of *GAPDH*, *B2M*, and *LDHA* -mRNAs was assessed by nCounter NanoString technology. The different HepG2 derived cell-lines are indicated on the X-axis, and the fold change of mRNA (log2 transformed) is indicated on the Y-axis. *PPARγ*-mRNA were decreased in both PPARγ-KD cell-lines compared to the GFP cells (by ~4–5 fold), and compared to ‘U’ cells (~3–4 fold).

We analyzed the levels of *PPARγ*-mRNA in both PPARγ knockdown cell-lines, PPARγ-KD1 and PPARγ-KD2 (four biological replicates each), by the NanoString nCounter method. We evaluated the reduction in *PPARγ* expression, and found that the levels of *PPARγ*-mRNA in PPARγ-KD1 were reduced to 23% and 36% compared to the GFP and the untransduced control HepG2 cell-lines, respectively (Fig 1). These results reproduced our previous data using TaqMan qRT-PCR that demonstrated >2-fold lower *PPARγ*-mRNA levels upon PPARγ knockdown in this cell-line [32]. Similarly, the levels of *PPARγ*-mRNA in PPARγ-KD2 cell-line were decreased to 17% and 26% relative to the GFP and the untransduced controls (Fig 1). For validation we repeated *PPARγ*-mRNA expression analysis using qRT-PCR (S1 Fig).

**Fig 2:**
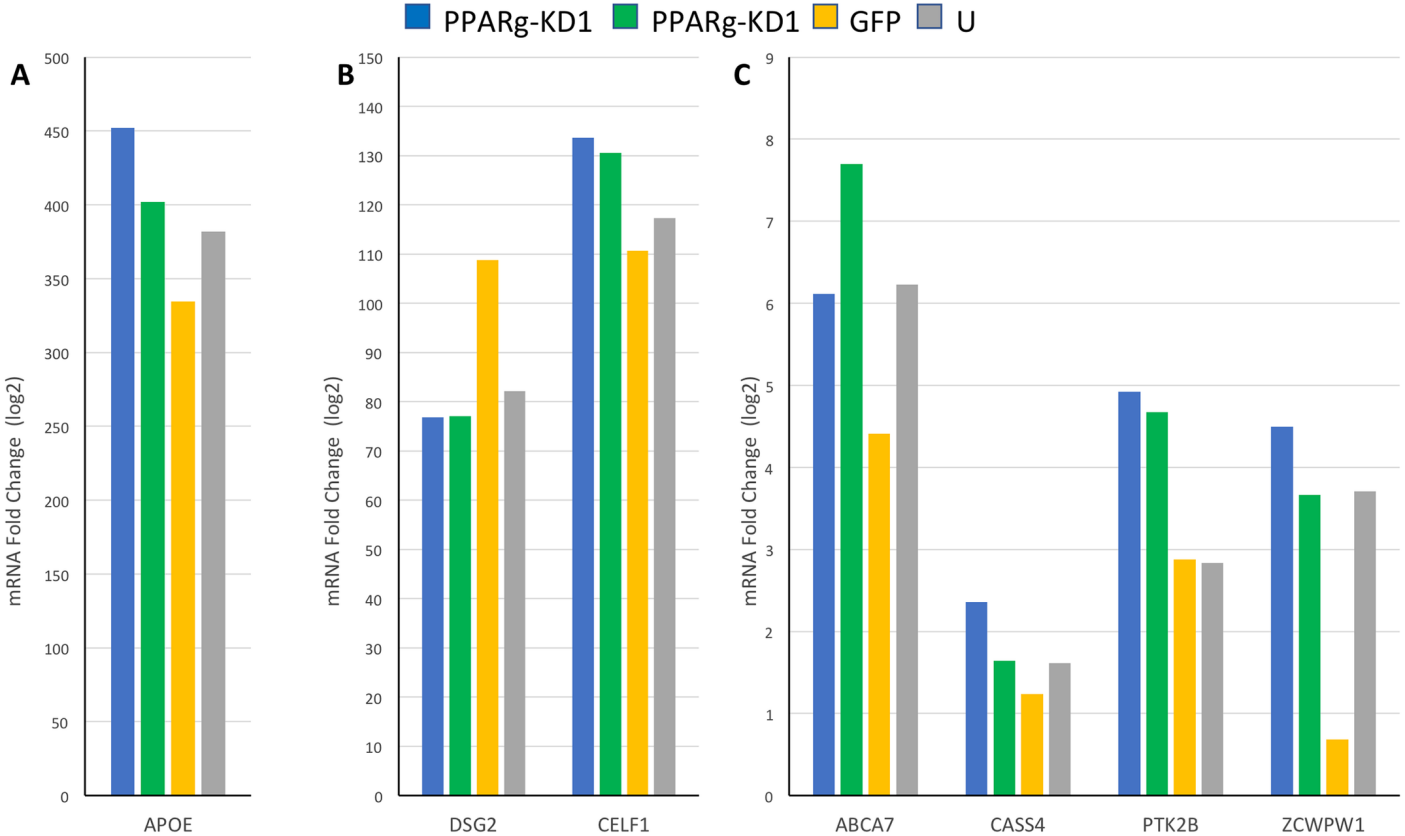
The effect of *PPARγ* knockdown in HepG2 cells on mRNA levels of LOAD-GWAS genes. RNA was extracted from four HepG2 derived cell-lines: PPARγ-KD1, PPARγ-KD2, GFP, and untransduced (U). For each cell-line, RNA samples from four biological replicates were pooled. The fold levels of (A) *APOE*-mRNA—represents highly expressed genes, (B) *DSG2*, and *CELF* -mRNA—represent medium expressed genes, and (C) *ABCA7*, *CASS4*, *PTK2B*, and *ZCWPW* -mRNA—represent lower expressed genes, compared to the geometric mean of *GAPDH*, *B2M*, and *LDHA* -mRNAs were assessed by nCounter NanoString technology. The different HepG2 derived cell-lines are indicated on the X-axis, and the fold change of mRNA (log2 transformed) is indicated on the Y-axis. The expression levels of *ABCA7*, *APOE*, *CASS4*, *CELF1*, *PTK2B*, and *ZCWPW1* -mRNAs were increased (A, B, C), and that of *DSG2*-mRNA (B) was decreased in PPARγ-KD1 and -KD2 cells compared to GFP and U cells.

Next, we examined the expression of twenty-four LOAD susceptibility genes inferred by the proximity to the most significant LOAD-associated SNPs (Table 1). Ten genes resulted in counts below the background threshold for all four HepG2 derived cell-lines and were considered as not expressed in our system (Table 1). We assessed the fold-change in mRNA levels for all fourteen remaining genes expressed in our HepG2 cells, by comparing the mRNA levels in PPARγ-KD1 and PPARγ-KD2 cell-lines relative to the GFP cell-line, using a pool of 4 biological replicates for each cell-line. A total of seven genes were found to be affected by *PPARγ* knockdown and demonstrated a consistent direction of the effect on mRNA levels, reproducible in both PPARγ-KD1 and PPARγ-KD2 cell-lines, that was 10% or more (Table 2, Fig 2). We observed that knockdown of *PPARγ* led to either increase or decrease in mRNA expression levels. Six genes were upregulated in PPARγ-KD cells: ATP binding cassette subfamily A member 7 (*ABCA7*), *APOE* (validated by qRT-PCR, S1 Fig, and consistent with our previous results [32]), Cas scaffolding protein family member 4 (*CASS4*), CUGBP Elav-like family member 1 (*CELF1*), protein tyrosine kinase 2 beta (*PTK2B*), and zinc finger CW-type and PWWP domain containing 1 (*ZWPW1*) (Table 2, Fig 2). Only one gene, desmoglein 2 (*DSG2*), was downregulated in the *PPARγ*-KD cell-lines compared to GFP cell-line (Table 2, Fig 2). These results reinforced the broad role of PPARγ as transcriptional activator and repressor [33], and demonstrated the effects on down- and up-regulation for LOAD-associated genes. Two genes, *BIN1* and *EPHA1*, showed opposites effects in PPARγ-KD1 compared to PPARγ-KD2. While *BIN1* and *EPHA1* mRNA levels were higher in PPARγ-KD1 relative to the GFP cell-line, they were lower in PPARγ-KD2. Five genes, *CD2AP*, *FERMT2*, *INPP5D*, *PICALM* and *SORL1*, showed no effect (<10%) on expression levels upon *PPARγ* knockdown.

**Table 2:** *PPARγ* knockdown effects on mRNA levels of LOAD-genes.

Gene	PPARγ-KD1 mRNA fold change	PPARγ-KD2 mRNA fold change	GO terms: Biological Process (GO ID)
**ABCA7**	1.39	1.75	transport (GO:0006810) phagocytosis (GO:0006909) aminophospholipid transport (GO:0015917)
**APOE**	1.35	1.20	response to dietary excess (GO:0002021) lipid metabolic process (GO:0006629) transport (GO:0006810) lipid transport (GO:0006869) cellular calcium ion homeostasis (GO:0006874) response to oxidative stress (GO:0006979) steroid metabolic process (GO:0008202) regulation of gene expression (GO:0010468) low-density lipoprotein particle remodeling (GO:0034374) lipoprotein metabolic process (GO:0042157) lipoprotein biosynthetic process (GO:0042158) vasodilation (GO:0042311) positive regulation of catalytic activity (GO:0043085) artery morphogenesis (GO:0048844) negative regulation of inflammatory response (GO:0050728) maintenance of location in cell (GO:0051651) lipid homeostasis (GO:0055088) cardiovascular system development (GO:0072358) negative regulation of triglyceride metabolic process (GO:0090209) regulation of plasma lipoprotein particle levels (GO:0097006) cellular oxidant detoxification (GO:0098869)
**CASS4**	1.91	1.33	cell adhesion (GO:0007155)
**CELF1**	1.21	1.18	spermatid development (GO:0007286) RNA splicing (GO:0008380) mRNA processing (GO:0006397) negative regulation of translation (GO:0017148) positive regulation of multicellular organism growth (GO:0040018)
**DSG2**	0.71	0.71	cell adhesion (GO:0007155) homophilic cell adhesion via plasma membrane adhesion molecules (GO:0007156) response to progesterone (GO:0032570) maternal process involved in female pregnancy (GO:0060135)
**PTK2B**	1.71	1.62	*MAPK cascade (GO:0000165) response to reactive oxygen species (GO:0000302) oocyte maturation (GO:0001556) response to hypoxia (GO:0001666) adaptive immune response (GO:0002250) immune system process (GO:0002376) regulation of leukocyte chemotaxis (GO:0002688) response to osmotic stress (GO:0006970) actin filament organization (GO:0007015) cell adhesion (GO:0007155)
**ZCWPW1**	6.56	5.34	None

Fold change in mRNA levels for each *PPARγ* knockdown cell-line, PPARγ-KD1 and PPARγ-KD2, were calculated relative to the control GFP cell-line. GO terms listed here include the subset using the filter for evidence used in automatic assertion (Inferred from Electronic Annotation (IEA)).

*Showing the first 10 out of 54 GO IDs filtered using IEA evidence.

## Discussion

Using NanoString technology, we analyzed the effect of *PPARγ* knockdown on expression of twenty-four LOAD-associated genes in human hepatocyte-derived cell-lines and demonstrated that PPARγ regulates the expression of seven LOAD-associated genes, including *APOE*. It has been demonstrated that PPARγ can both activate and repress transcription, in ligand-dependent or independent manners, via binding to coactivators or corepressors, respectively [34]. Here we showed that *PPARγ* knockdown resulted in upregulation of six genes (*ABCA7*, *APOE*, *CASS4*, *CELF1*, *PTK2B*, and *ZCWPW1*) and downregulation of one gene (*DSG2*). Annotation of these genes using GO terms for biological processes (Table 2) indicated the involvement of these genes in diverse biological processes including pathways related to lipid metabolism, immune function, and cellular stress response, suggesting a role for PPARγ in these aspects of LOAD etiology.

PPARγ is the most extensively studied member of the PPARs family; it is well known for its role in peripheral metabolism, and has been implicated in the pathology of numerous diseases including diabetes, stroke, cancer, and obesity [18]. Accumulating evidence has also suggested the involvement of PPARγ in the pathogenesis of various disorders of the central nervous system (CNS) and several brain neuropathologies including LOAD [16, 17, 19, 35]. PPARγ exhibits a wide range of activities related to Alzheimer’s pathology. Studies using animal models of Alzheimer’s suggested that PPARγ exerts direct and indirect effects on Aβ metabolism (reviewed in [35]). Furthermore, the Pro12Ala mutation in PPARγ was associated with LOAD risk and age-of-onset, however, other studies failed to detect any significant association between the Ala12 variant and the genetic risk of LOAD [36]. Nonetheless, it was reported that Ala12 carriers showed an increased risk of cognitive decline than non-carriers among diabetic patients [36, 37]. Here we found that out of fourteen LOAD-associated genes expressed in our cellular system, seven are regulated by *PPARγ*. Our study further strengthened the link between PPARγ and neurodegeneration, in particular the development of LOAD. Collectively, our research supports the potential beneficial impact of PPARγ agonists for ameliorating LOAD-related phenotypes, reinforcing the concept that PPARγ agonists may represent an attractive class of drugs for preventing or delaying the onset of LOAD. However, it is important to note that one limitation of this study is the cellular system, as it derived from hepatocytes and does not represent brain cell types relevant to LOAD. Therefore, further investigations using the three major brain cell-types implicated in LOAD, neurons, astrocytes, and microglia, are warranted. These follow up studies will provide insight into the regulatory impact of *PPARγ* on the complete repertoire of LOAD-associated genes, in the context of the intracellular environments of cell-types involved in LOAD pathogenesis.

PPARγ governs metabolism, immune response, and other biological processes that are also critical for the resilience of human tissues and organs through life-span and aging ‘physiological failure’. We hypothesize that the possible master role of PPARγ in tissue resilience underlies its involvement in distinct diseases from cancer to LOAD. Here we found the effect of PPARγ on genes that were implicated in both LOAD and cancer. The LOAD-associated genes, cas scaffolding protein family member 4 (*CASS4*) and protein tyrosine kinase 2 beta (*PTK2B*), have been studied primarily for their roles in the directly cancer-relevant processes of migration and survival signaling. *CASS4* and *PTK2B* act as interacting partners regulating oncogenesis and metastasis, and are known to be active in the brain during development and in cancer [38]. In addition, *CELF1* has been associated with certain types of cancer [39, 40], and expression of *ABCA7* significantly increased in ovarian carcinoma [41]. It has been reported that cancer survivors are at reduced risk for LOAD, and that people with LOAD may be at a reduced risk of developing cancer [42, 43]. The identification of genetic associations of cancer genes with LOAD risk suggested the intriguing hypothesis of mechanistic overlap between cancer and Alzheimer’s disease; moreover, our findings that four of these common genes are co-regulated by PPARγ suggest a possible role for PPARγ in the interplay between LOAD and cancer.

GWAS identified over twenty tagging SNPs associated with LOAD—however, the exact target genes that contribute directly to the disease within each of the LOAD associated genomic regions have yet to be identified. For example, there are about ten genes in the region defined by the LOAD associated-SNPs that has been inferred to as *ZCWPW1*. Here, we described the expression analysis of LOAD GWAS genes inferred by the proximity to the most significantly associated SNPs. In-depth exploration of the regulatory role of PPARγ in the context of LOAD warrants further investigations evaluating an inclusive and unbiased list of genes that are positioned within +/-1Mb (a range proposed as a range of LD for mapping disease genes [44, 45]) surrounding the genome-wide significant associated-SNPs.

## Supporting information

S1 Fig Validation of the mRNA expression changes using TaqMan based qRT-PCR assays. RNA was extracted from four HepG2 derived cell-lines: PPARγ-KD1, PPARγ-KD2, GFP, and untransduced (U). The levels of (A) *PPARγ*-mRNA, and (B) *APOE*-mRNA, relative to the geometric mean of *GAPDH-* and *PPIA* -–mRNAs, were assessed by real-time PCR and analyzed by the 2^-ΔΔCt^ method. The different HepG2 derived cell-lines are indicated on the X-axis, and the fold change of mRNA (log2 transformed) is indicated on the Y-axis. The values presented here are means levels±SEM of 4 replicates. Student’s t-test analysis was used to determine significant differences. (A) *PPARγ*-mRNA were significantly decreased (p<0.0001) in PPARγ-KD1 and PPARγ-KD2 cells compared to GFP cells and U cells. (B) *APOE*-mRNA were significantly increased in PPARγ-KD1 (p = 0.0003) and PPARγ-KD2 (p = 0.03) cells compared to GFP cells.
(DOCX)

S1 Table Genes analyzed with NanoString. Genes analyzed using NanoString technology, including twenty-four LOAD-associated genes, PPARγ, and the housekeeping genes B2M, GAPDH, and LDHA. Gene Accession numbers and the region and sequence targeted by each probe are described.
(XLSX)
