# Supplementary material for: Probing the role of PPARγ in the regulation of late-onset Alzheimer’s disease-associated genes
Source: PLoS One. 2018 May 3;13(5):e0196943. doi: 10.1371/journal.pone.0196943 (PMC5933777; doi:10.1371/journal.pone.0196943)

**Figure S1. Validation of the mRNA expression changes using TaqMan based qRT-PCR assays**

RNA was extracted from three HepG2 derived cell-lines: PPARγ KD1, PPARγ KD2, GFP, and untransduced (U). The levels of (A) *PPARγ*-mRNA and (B) *APOE*-mRNA relative to the geometric mean of *GAPDH-* and *PPIA* -mRNAs were assessed by real-time PCR and were analyzed by the 2^-ΔΔCt^ method. The different HepG2 derived cell-lines are indicated on the X-axis, and the fold change of mRNA (log2 transformed) is indicated on the Y-axis. The values presented here are means levels$\pm$SEM of 4 replicates. Student’s t-test analysis was used to determine significant differences. (A) *PPARγ*-mRNA were significantly decreased (p<0.0001) in PPARγ KD1 and PPARγ KD2 cells compared to the GFP cells and the U cells. (B) *APOE*-mRNA were significantly increased in PPARγ KD1 (p=0.0003) and PPARγ KD2 (p=0.03) cells compared to the GFP cells.

**A**


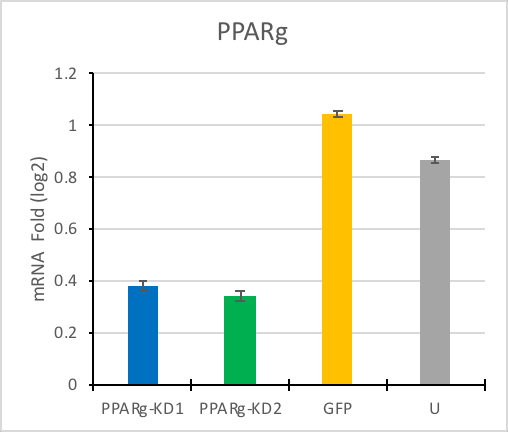


**B**


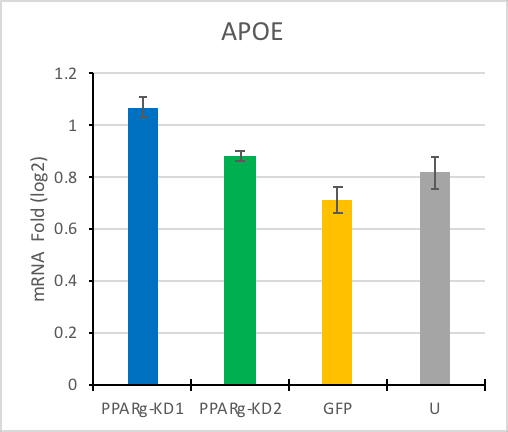

Supplement: S1 Fig — RNA was extracted from four HepG2 derived cell-lines: PPARγ-KD1, PPARγ-KD2, GFP, and untransduced (U). The levels of (A) PPARγ-mRNA, and (B) APOE-mRNA, relative to the geometric mean of GAPDH- and PPIA -–mRNAs, were assessed by real-time PCR and analyzed by the 2-ΔΔCt method. The different HepG2 derived cell-lines are indicated on the X-axis, and the fold change of mRNA (log2 transformed) is indicated on the Y-axis. The values presented here are means levels±SEM of 4 replicates. Student’s t-test analysis was used to determine significant differences. (A) PPARγ-mRNA were significantly decreased (p<0.0001) in PPARγ-KD1 and PPARγ-KD2 cells compared to GFP cells and U cells. (B) APOE-mRNA were significantly increased in PPARγ-KD1 (p = 0.0003) and PPARγ-KD2 (p = 0.03) cells compared to GFP cells. (DOCX) [file pone.0196943.s001.docx]
